# Study on the mechanism of action of Chaihu Guizhi Ganjiang Decoction for the treatment of slow transit constipation combined with depression based on network pharmacology and molecular docking

**DOI:** 10.1097/MD.0000000000049136

**Published:** 2026-06-05

**Authors:** Zixu Zhao, Chaolun Zhu, Lingfei Meng, Shuangxi Zhang

**Affiliations:** a Department of Anorectal, The First Affiliated Hospital of Henan University of Chinese Medicine, Zhengzhou, China; b Henan University of Chinese Medicine, Zhengzhou, China; c The First Affiliated Hospital of Henan University of Chinese Medicine, Zhengzhou, China.

**Keywords:** Chaihu Guizhi Ganjiang Decoction, depression, molecular docking, network pharmacology, slow transit constipation

## Abstract

Based on network pharmacology and molecular docking technology, this study investigated the mechanism of action of Chaihu Guizhi Ganjiang Decoction (CGGD) in the treatment of slow transit constipation (STC) combined with depression. The Traditional Chinese Medicine System Pharmacology Database Analysis Platform and BATMAN-TCM databases were utilized to screen the active ingredients and target proteins of CGGD. The keywords “slow transit constipation” and “depression” were used to query the GEO, GeneCards, and OMIM databases to obtain the disease targets; the intersection targets of the TCM formula and the disease were taken to analyze the protein interactions among the target genes, and to construct the protein–protein interaction network diagram and the “drug-ingredient-target-disease” interaction network. Gene Ontology functional enrichment and Kyoto Encyclopedia of Genes and Genomes pathway were used to analyze the enrichment, and molecular docking was used to further confirm the interactions between core ingredients and key targets. Identified 138 active ingredients, 141 target proteins, and 17,110 disease targets. Analysis yielded 10 core ingredients, including quercetin, kaempferol, wogonin, and baicalein, alongside 10 key targets such as estrogen receptor 1, epidermal growth factor receptor, caspase-3, and B-cell lymphoma-2. Molecular docking studies demonstrated strong binding affinities between core ingredients and key targets. Enrichment analysis identified 440 biological processes and 131 pathways. CGGD primarily intervenes in STC combined with depression through pathways including the phosphatidylinositol 3-kinase-Akt signaling pathway, the mitogen-activated protein kinase signaling pathway, and the apoptosis pathway. Through an integrated network pharmacology approach, this study describes the synergistic effects of multiple ingredients, targets, and pathways of CGGD in the treatment of STC combined with depression.

## 1. Introduction

Constipation is a common gastrointestinal dysfunction disease. According to its causes, it can be divided into 2 categories: functional constipation and organic constipation, of which functional constipation is more common. Functional constipation can be subdivided into slow transit constipation (STC), outlet obstruction constipation, and mixed constipation according to its pathophysiologic mechanism.^[1,2]^ STC is a functional disorder disease in which intestinal contents remain in the intestinal tract for a long period of time due to the reduction of colonic transport capacity and the slowing down of transport speed,^[3]^ and its main clinical manifestations include dry and hard feces, difficulty in defecation, and a decrease in the frequency of defecation.^[4]^ In recent years, due to the increase in social pressure and the change in diet structure, the incidence of STC has shown a rising trend year by year,^[5]^ with a global incidence rate of 15% and an incidence rate of about 8% in China.^[6,7]^ Long-term constipation may not only induce intestinal tumors and cardiovascular and cerebrovascular diseases, but also lead to serious complications such as intestinal obstruction and intestinal perforation, or even threaten the safety of life in severe cases.^[8]^ It is noteworthy that the physiological functions of the gut and mental-psychological states form a tightly regulated network via the “gut-brain axis.” On the one hand, metabolites from the gut microbiota can enter the circulation through the intestinal mucosal barrier, regulating central nervous system inflammation and neurotransmitter balance. On the other hand, psychological stress can conversely inhibit intestinal motility and disrupt the stability of the gut microbiota. This bidirectional regulation constitutes the core pathological basis for the co-morbidity of STC and depression.^[9,10]^ Research^[11]^ has shown that the prevalence of STC combined with depression is increasing year by year, which not only brings great pain to patients and their families but also brings a serious economic burden to society. Currently, western medicine treats STC mainly with chemical drugs such as laxatives and prokinetic agents, but it is easy to produce laxative dependence, and the long-term efficacy is not good,^[12]^ and to a certain extent, it also aggravates the patient’s depressive mood. For patients with STC combined with depressive symptoms, in addition to the necessary drug treatment, psychological interventions are also needed, often combined with antidepressant drugs, but most of these drugs have the side effect of aggravating constipation.^[13]^ Therefore, identifying novel therapeutic strategies is particularly important.

The active constituents within natural botanicals, possessing enhanced biocompatibility with the human physiological environment, the regulatory potential of “multiple targets per molecule,” and synergistic properties through multi-ingredient interactions, render them a significant research focus for treating complex diseases. Traditional Chinese medicinal formulas inherently embody this advantage. Functioning as “functional complexes” of natural plant constituents, their “monarch, minister, assistant, and envoy” formulation principles fundamentally achieve multi-pathological coverage through complementary interactions among multiple ingredients. This approach aligns profoundly with the concept of “holistic regulation” central to contemporary biomedicine. A metabolite complex composed of multiple herbal extracts can exert synergistic effects through the combined action of its various constituents, simultaneously delivering multiple benefits, including antioxidant and anti-inflammatory properties, alongside regulation of intestinal motility and neurological function.^[14,15]^ Chaihu Guizhi Ganjiang Decoction (CGGD) originates from the classical Chinese medical text “Shang Han Lun.” It is composed of 7 traditional Chinese medicines: Chaihu, Guizhi, Ganjiang, *Scutellaria baicalensis*, Gualougen, Calcined Oyster, and Zhigancao. It is widely used in the treatment of gastrointestinal and neuropsychiatric diseases.^[16,17]^ However, its molecular mechanisms in managing STC combined with depression remain unexplored.

The application of network pharmacology integrates emerging interdisciplinary fields such as systems biology and bioinformatics, establishing itself as a core tool for deciphering the multi-target mechanisms underlying complex diseases.^[18]^ It offers novel perspectives and methodologies for elucidating intricate action mechanisms.^[19]^ By integrating information from multiple databases, it enables rapid screening of core drug ingredients, prediction of key targets and signaling pathways, and effectively addresses the complex “multi-ingredient, multi-target, multi-pathway” challenges inherent in traditional Chinese medicine formulations. In studies of complex diseases such as metabolic disorders and tumors, network pharmacology has successfully validated the synergistic regulatory mechanisms of plant-derived drugs, providing scientific support for the modern interpretation of traditional medicines.^[20]^ This characteristic of network pharmacology aligns with the theoretical frameworks of TCM’s “holistic view” and “syndrome differentiation and treatment,” establishing it as a pivotal bridge connecting traditional Chinese medicine with modern biomedicine.^[21]^ Molecular docking is a theoretical simulation method for studying intermolecular interactions and predicting their binding patterns and affinities, and has become a crucial technique in the field of computer-aided drug research.

Based on this, the present study employs network pharmacology and molecular docking techniques to systematically elucidate the core ingredients, key targets, and synergistic regulatory pathways of CGGD. This aims to reveal its mechanism of action in treating STC combined with depression, thereby providing a theoretical foundation for subsequent research and clinical application of CGGD.

## 2. Experimental methods

### 2.1. Active ingredient screening and target prediction

Utilized the Traditional Chinese Medicine System Pharmacology Database Analysis Platform (TCMSP,^[22,23]^ https://www.tcmsp-e.com/tcmsp.php), chemical ingredient information was retrieved for the constituents of CGGD: Chaihu, Guizhi, Ganjiang, *S Baicalensis*, Gualougen, and Zhigancao in CGGD. Active ingredients were screened based on oral bioavailability ≥ 30% and drug-like properties ≥ 0.18, identifying active ingredients and their corresponding target proteins. For herbal medicines not retrieved from the TCMSP database, the BATMAN-TCM database^[24]^ (http://bionet.ncpsb.org.cn/batman-tcm/) was employed to supplement the search. A similarity score cutoff ≥ 20 and *P* < .05 to identify the active ingredients and potential target proteins of calcined oyster. The drug-target proteins were then converted to their corresponding target gene names via the UniProt database (https://www.uniprot.org/).

### 2.2. Target screening for STC combined with depression

Searched and filtered the GEO database (https://www.ncbi.nlm.nih.gov/geo/), GeneCards database (https://www.genecards.org/), and OMIM database (https://omim.org/) using the keywords “slow transit constipation” and “depression” to obtain the currently known targets related to STC combined with depression.

### 2.3. Screening for CGGD and STC combined with depression common targets

The obtained CGGD drug target genes were mapped to STC combined with depression disease target genes, and the common targets between them were screened, and Venny plots of the potential targets of CGGD for STC combined with depression were obtained using the Venny online platform (https://bioinfogp.cnb.csic.es/tools/venny/).

### 2.4. Constructing protein interaction analysis network between target genes protein–protein interaction (PPI) and key targets prediction

Imported the intersecting gene targets into the STRING 12.0 database (https://cn.string-db.org/) for PPI analysis among target genes. Specify the species as “*Homo sapiens*,” set the minimum interaction score to “0.400,”^[25]^ and retain all other parameters at their defaults to generate the PPI network diagram. The PPI network diagram was imported into Cytoscape 3.10.0 software. Network topology analysis was performed using the Cytoscape 2.2 plugin, evaluating degree centrality, betweenness centrality, and closeness centrality. The target genes were then sorted in descending order by degree value, with the top 10 genes selected as key targets.

### 2.5. Constructing a “drug-ingredient-target-disease” network diagram

Imported drugs, ingredients, targets, and diseases into an Excel spreadsheet to establish corresponding relationships, then utilized Cytoscape 3.10.0 to construct a “drug-ingredient-target-disease” network diagram. Within this diagram, nodes represent drug, ingredient, target, and disease names, whilst edges denote interactions between drugs and ingredients, ingredients and targets, and diseases and targets. Utilized the built-in “Network Analyser” plugin to analyze the network diagram; the top 10 active ingredients by degree value were selected as the core ingredients of the CGGD.

### 2.6. Gene Ontology (GO) and Kyoto Encyclopedia of Genes and Genomes (KEGG) Pathway Enrichment Analysis

The core targets were enriched and analyzed through the DAVID database^[26]^ (https://davidbioinformatics.nih.gov/), and the results were imported into the microbiology platform (http://www.bioinformatics.com.cn/) for bubble mapping.

### 2.7. Molecular docking of drug core ingredients to targets

Based on the degree values, selected the top 4 core ingredients and key targets for molecular docking. This approach serves 2 purposes: firstly, ingredients and targets with higher rankings demonstrate broader connectivity and more prominent core regulatory functions; secondly, it validates that these core ingredients and key targets possess greater scientific value and translational significance. Download the 2D structures of core ingredient ligands from PubChem (https://pubchem.ncbi.nlm.nih.gov/) as SDF files, then convert them to mol2 format using ChemBio3D Ultra 14.0. Download the 3D structures of key targets from the PDB database (https://www.rcsb.org/), remove water molecules and small-molecule ligands, and save them in PDB format. AutoDock Tools 1.5.7 was employed to convert ligands and proteins into pdbqt format and define binding pockets. AutoDock Vina performed molecular docking calculations, with PyMOL subsequently used for visualization analysis. Binding strength was assessed by combining the docking scores, where a binding energy below −5.0 kcal/mol indicates favorable binding activity between the ligand and receptor protein.^[27]^

## 3. Results

### 3.1. CGGD active ingredient screening and target prediction

Based on the TCMSP database and BATMAN-TCM database, we obtained the composition information of the core drugs “Chaihu, Guizhi, Ganjiang, *S baicalensis*, Gualougen, Calcined Oyster, and Zhigancao” as well as the predicted potential targets. There were 17 active ingredients and 633 targets for Chaihu, 7 active ingredients and 165 targets for Guizhi, 5 active ingredients and 139 targets for Ganjiang, 36 active ingredients and 629 targets for *S baicalensis*, 2 active ingredients and 5 targets for Gualougen, 5 active ingredients and 27 targets for Calcined Oyster, and 92 active ingredients and 2032 targets for Zhigancao. All the ingredients and targets were integrated, and the duplicate values were deleted; finally, 138 active ingredients and 141 drug targets were obtained (Table 1, Tables S1 and S2, Supplemental Digital Content).

**Table 1:** Information of some active ingredients of CGGD.

MOL ID	MOL name	OB	DL	Source drugs
MOL000098	quercetin	46.43	0.28	Chaihu, Zhigancao
MOL000422	kaempferol	41.88	0.24	Chaihu, Zhigancao
MOL000354	isorhamnetin	49.6	0.31	Chaihu, Zhigancao
MOL000358	beta-sitosterol	36.91	0.75	Chaihu, Ganjiang, *Scutellaria baicalensis*
MOL000359	sitosterol	36.91	0.75	Guizhi, Ganjiang, *Scutellaria baicalensis*, Zhigancao
MOL000449	Stigmasterol	43.83	0.76	Chaihu, *Scutellaria baicalensis*
MOL000073	ent-Epicatechin	48.96	0.24	Guizhi, *Scutellaria baicalensis*
MOL001645	Linalyl acetate	42.1	0.2	Chaihu
MOL002776	Baicalin	40.12	0.75	Chaihu
MOL004609	Areapillin	48.96	0.41	Chaihu
MOL001736	(−)-taxifolin	60.51	0.27	Guizhi
MOL000492	(+)-catechin	54.83	0.24	Guizhi
MOL004576	taxifolin	57.84	0.27	Guizhi
MOL002464	1-Monolinolein	37.18	0.3	Ganjiang
MOL002514	Sexangularetin	62.86	0.3	Ganjiang
MOL001689	acacetin	34.97	0.24	*Scutellaria baicalensis*
MOL000173	wogonin	30.68	0.23	*Scutellaria baicalensis*
MOL002714	baicalein	33.52	0.21	*Scutellaria baicalensis*
MOL004355	Spinasterol	42.98	0.76	Gualougen
MOL006756	Schottenol	37.42	0.75	Gualougen
MOL001484	Inermine	75.18	0.54	Zhigancao
MOL001792	DFV	32.76	0.18	Zhigancao
MOL000211	Mairin	55.38	0.78	Zhigancao
	Aluminum			Calcined Oyster
	Calcium Sulphate			Calcined Oyster
	Calcium Phosphate			Calcined Oyster

CGGD = Chaihu Guizhi Ganjiang Decoction, DL = drug-like properties, OB = oral bioavailability.

### 3.2. Obtain the targets of STC combined with depression disease

The keywords “slow transit constipation” and “depression” were entered into the GEO database, GeneCards database, and OMIM database, respectively. Among them, the GeneCards database filtered genes with a score ≥ 5,^[28]^ and the disease targets filtered from each database were collected and organized, ultimately resulting in 17,110 disease targets.

### 3.3. CGGD and STC combined with depression co-targets

**Figure 1.**
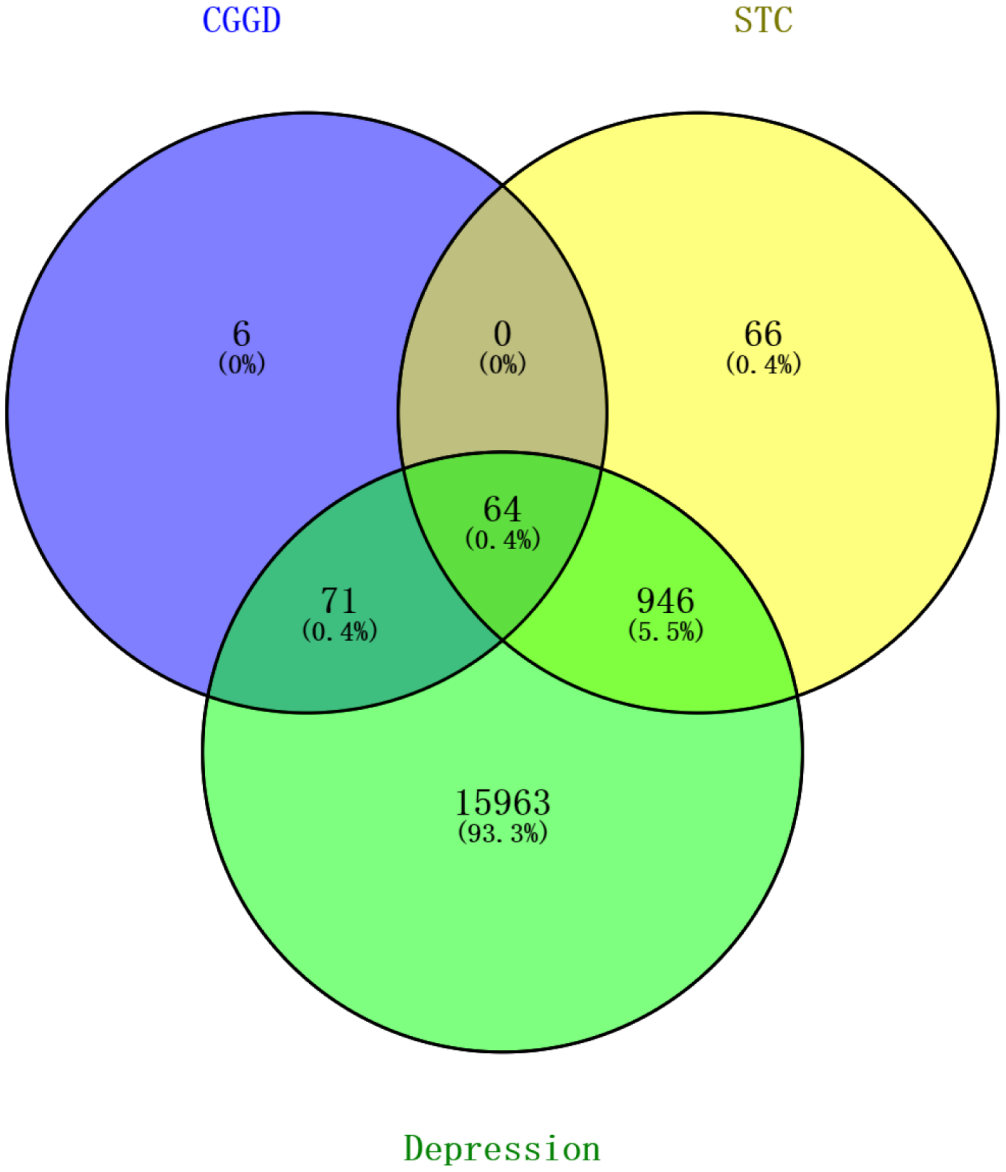
Drug targets and disease targets Venny Chart. CGGD = Chaihu Guizhi Ganjiang Decoction, STC = slow transit constipation.

Using the Venny online platform, CGGD drug targets were mapped to STC combined with depression disease targets to take the intersection. Sixty-four common targets were obtained, i.e., drugs were able to exert therapeutic effects on the disease through these 64 targets (Fig. 1, and Table S3, Supplemental Digital Content).

### 3.4. Protein interaction analysis network PPI and key gene prediction between target genes

**Figure 2.**
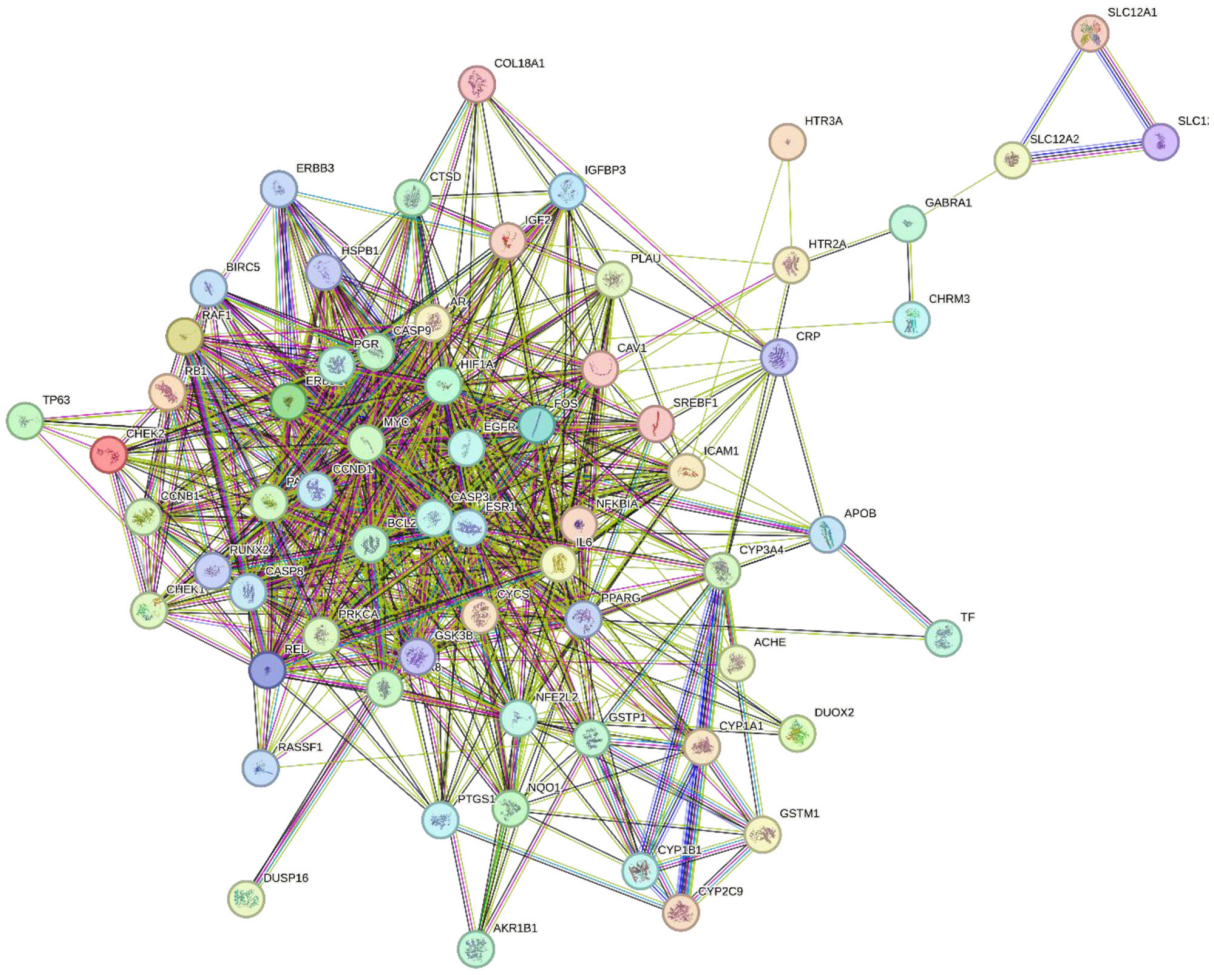
Intersecting genes PPI network diagram. PPI = protein–protein interaction.

**Figure 3.**
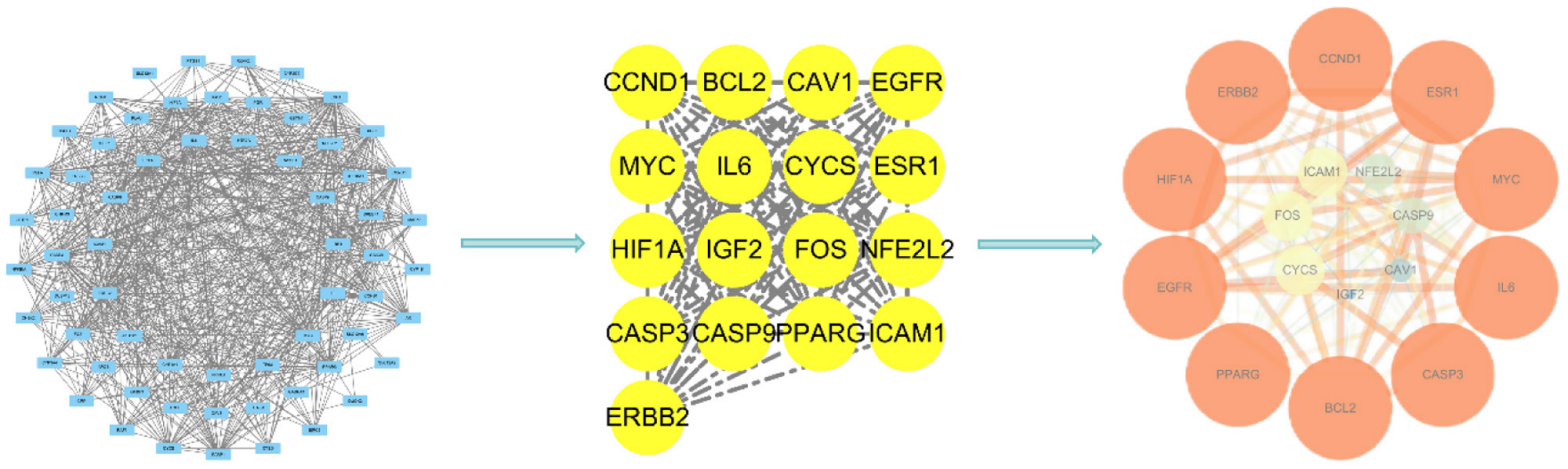
PPI network topology analysis core genes. PPI = protein–protein interaction.

Import the 64 intersecting targets from Section 3.3 into the STRING 12.0 database, and set the species to “*Homo sapiens*.” The string_interactions file was obtained and saved in TSV format, there are 64 nodes and 656 edges in the network, the average degree of the nodes is 20.5, in which the protein targets are shown through the nodes and PPIs are reflected through the edges between the 2, and the higher the number of neighboring nodes indicates that these proteins play a more significant role in the network (Fig. 2). The downloaded TSV file was imported into Cytoscape 3.10.0 software for topological property analysis to obtain the potential target protein interaction network for CGGD treatment of STC combined with depression, and the topological parameter analysis was performed using CentiScaPe 2.2, and Network Analyzer plug-in, according to the ingredients in the the network was sorted by the connectivity (Degree) value, in which estrogen receptor 1 (ESR1), epidermal growth factor receptor (EGFR), caspase-3 (CASP3), B-cell lymphoma-2 (BCL2), myelocytomatosis oncogene, interleukin 6 (IL6), hypoxia-inducible factor 1-alpha, G1/S-specific cyclin-D1, erb-B2 receptor tyrosine kinase 2, and peroxisome proliferator-activated receptor gamma were in the core position, suggesting that these genes might be the core genes of CGGD action in STC combined with depression (Table 2, Fig. 3).

**Table 2:** Top 10 protein degree values for core targets.

Target	Degree	Betweenness	Closeness
ESR1	47	210.5217399	0.011363636
EGFR	46	168.0302206	0.011235955
CASP3	45	139.941293	0.011111111
BCL2	44	100.9688068	0.010989011
MYC	44	128.1388381	0.010989011
IL6	43	182.7643788	0.010869565
HIF1A	42	99.94032615	0.010752688
CCND1	40	78.13709822	0.010526316
ERBB2	40	82.76766523	0.010526316
PPARG	37	85.07528759	0.010204082

BCL2 = B-cell lymphoma-2, CASP3 = caspase-3, CCND1 = G1/S-specific cyclin-D1, EGFR = epidermal growth factor receptor, ERBB2 = erb-B2 receptor tyrosine kinase 2, ESR1 = estrogen receptor 1, HIF1A = hypoxia-inducible factor 1-alpha, IL6 = interleukin 6, MYC = myelocytomatosis oncogene, PPARG = peroxisome proliferator-activated receptor gamma.

### 3.5. Constructing a “drug-ingredient-target-disease” network diagram

**Figure 4.**
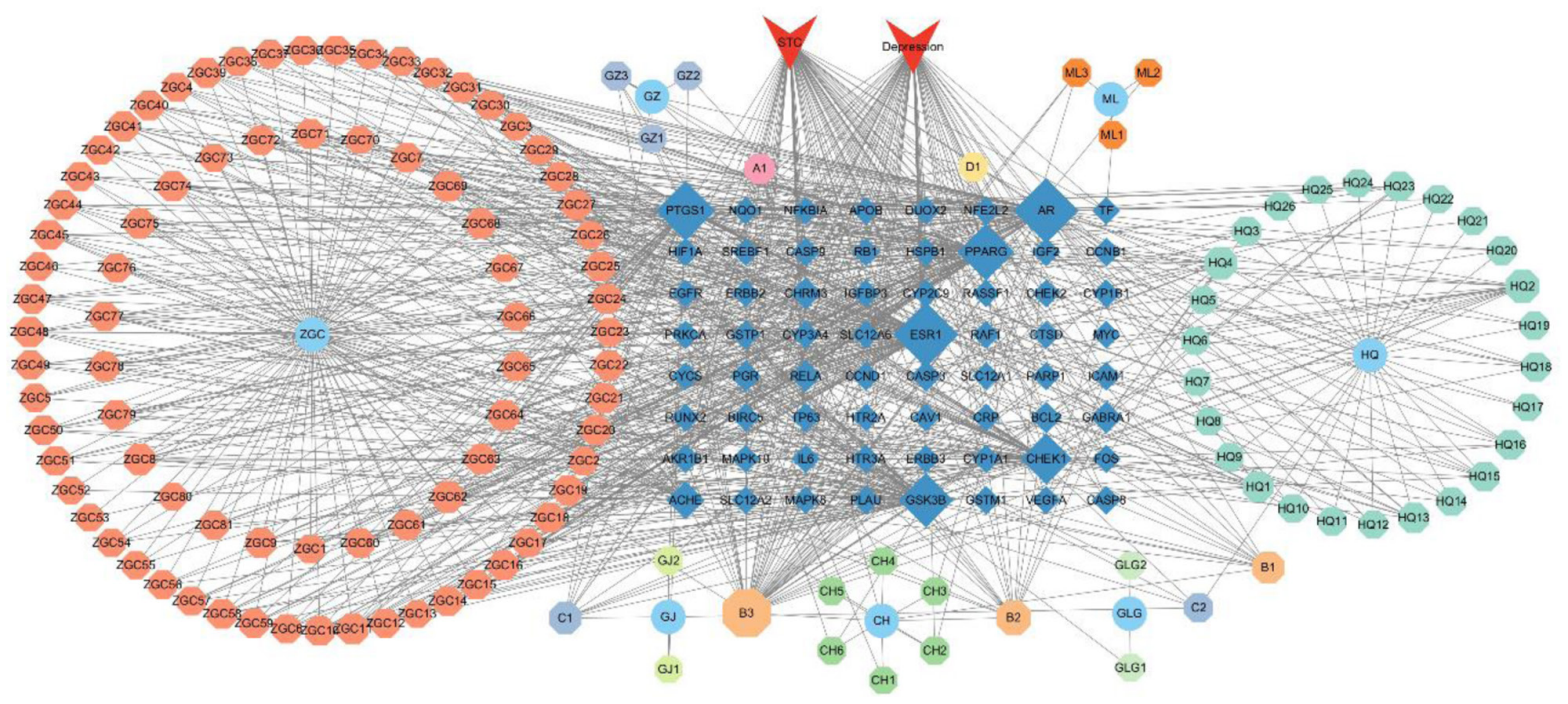
Drug-ingredient-target-disease network diagram.

The active ingredients, core targets, diseases and related targets of the TCM formulae were mapped to each other, and the “drug-ingredient-target-disease” network was constructed using Cytoscape 3.10.0 (Fig. 4), and the top 10 core ingredients with the highest degree were: quercetin, kaempferol, wogonin, baicalein, isorhamnetin, beta-sitosterol, licochalcone a, 7-methoxy-2-methyl isoflavone, naringenin, and acacetin, among which quercetin, the active ingredient, ranked first, suggesting that quercetin may play a major role in the pharmacological treatment of STC combined with depression.

### 3.6. GO and KEGG enrichment analysis of CGGD treatment of STC combined with depression

**Figure 5.**
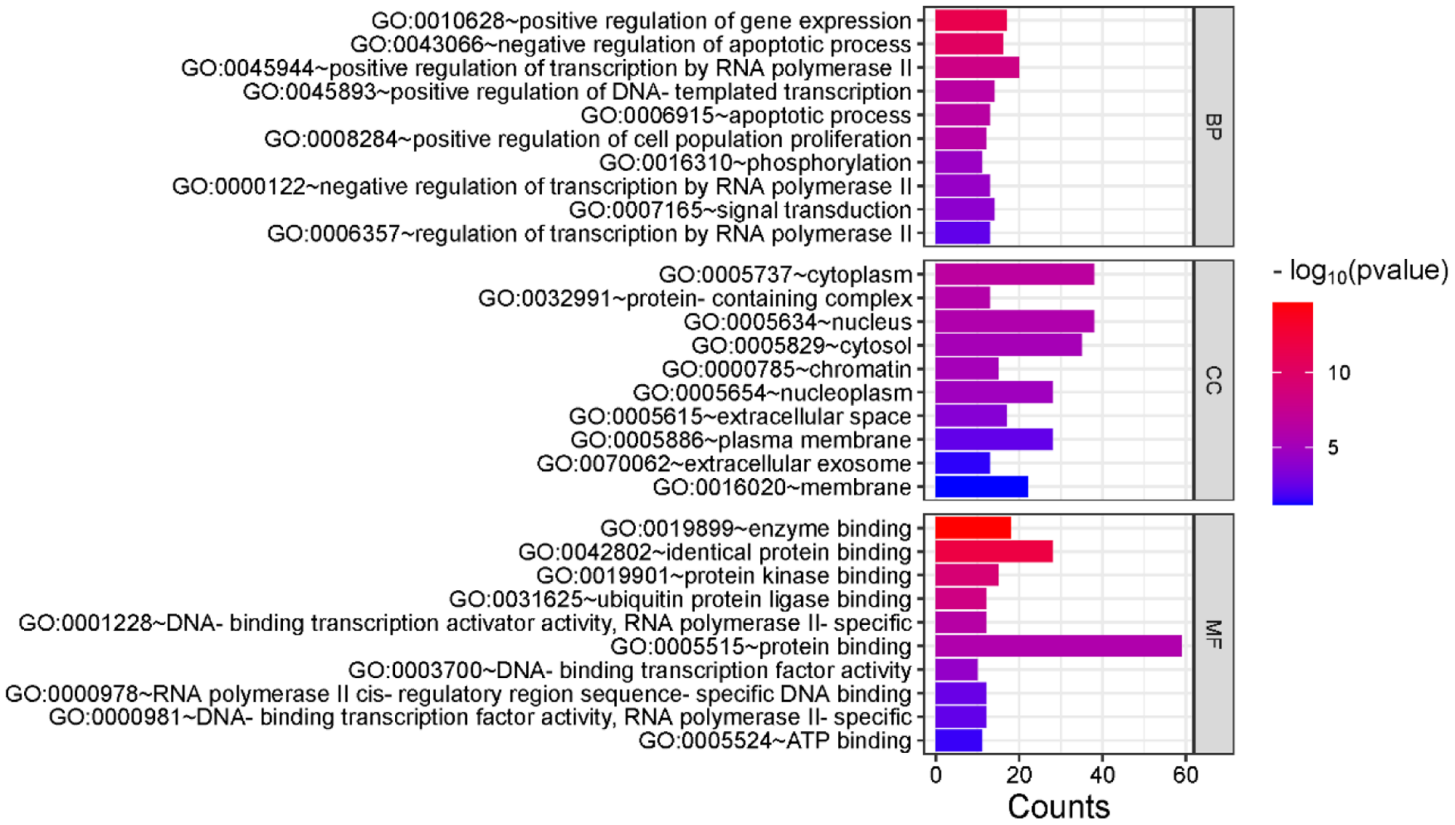
GO functional analysis. GO = Gene Ontology.

**Figure 6.**
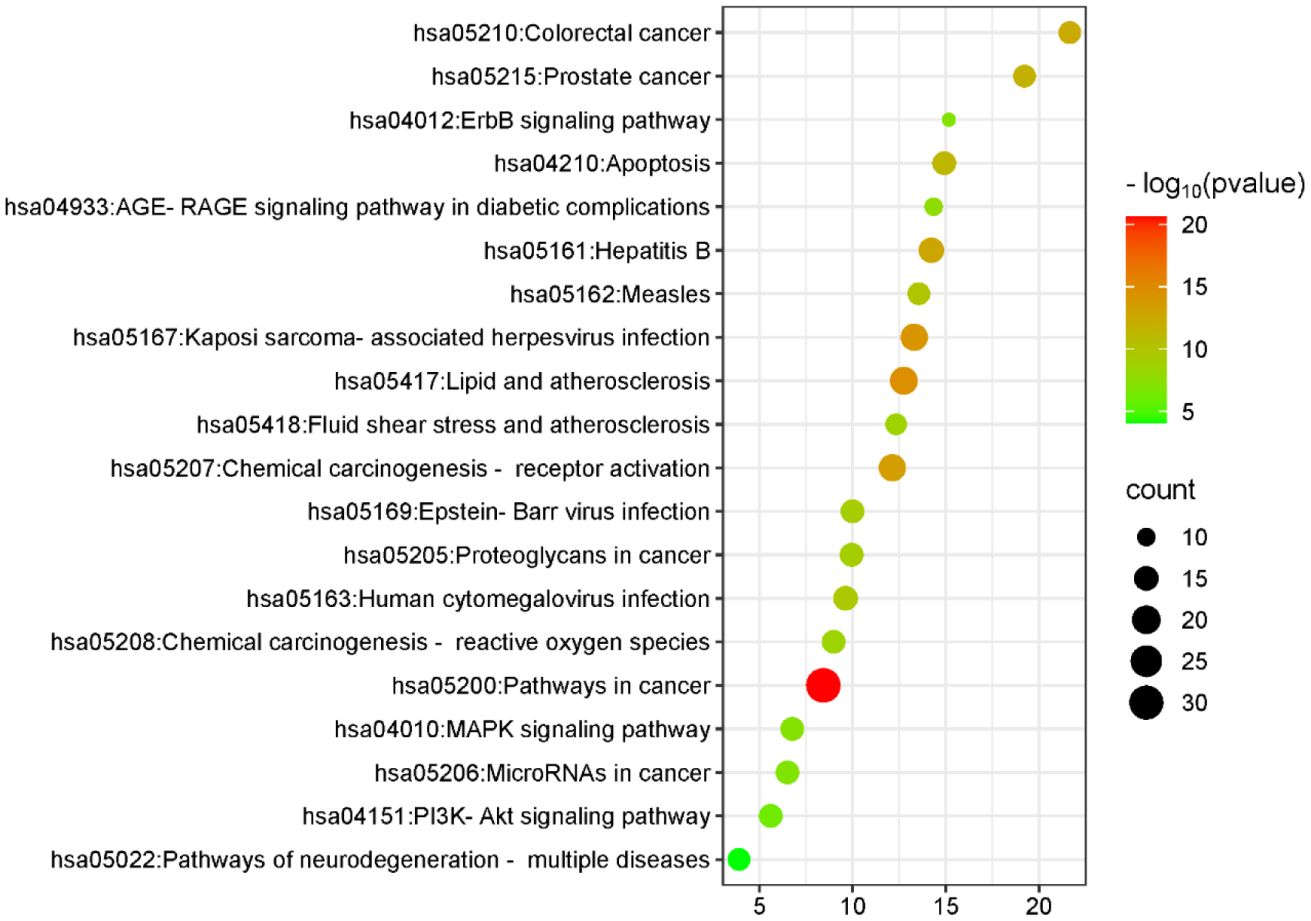
KEGG signaling pathway enrichment analysis. KEGG = Kyoto Encyclopedia of Genes and Genomes.

Bioinformatics analysis of GO biological process (BP) and KEGG pathway was performed on 64 drug-disease target genes using GO function enrichment and KEGG pathway, and the results of GO enrichment analysis can be categorized into 3 groups: BP, cellular component (CC), and molecular function. The BP involved in CGGD mainly involves the positive regulation of gene expression, the negative regulation of the apoptosis process, signaling, etc, and affects protein binding, enzyme binding, transcription factor activity, and specific protein binding, and plays a role in the nucleus, cytoplasm, and cytoplasmic membrane. Twenty entries were selected for bubble mapping (Fig. 5). KEGG-enriched pathway suggests that CGGD may be involved in the regulation of STC combined with depression through phosphatidylinositol 3-kinase (PI3K)-Akt signaling pathway, mitogen-activated protein kinase (MAPK) signaling pathway, ErbB signaling pathway, AGE-RAGE signaling pathway, apoptosis, cancer pathway, and so on (Fig. 6).

### 3.7. Results of docking the core ingredients with key target molecules

**Figure 7.**
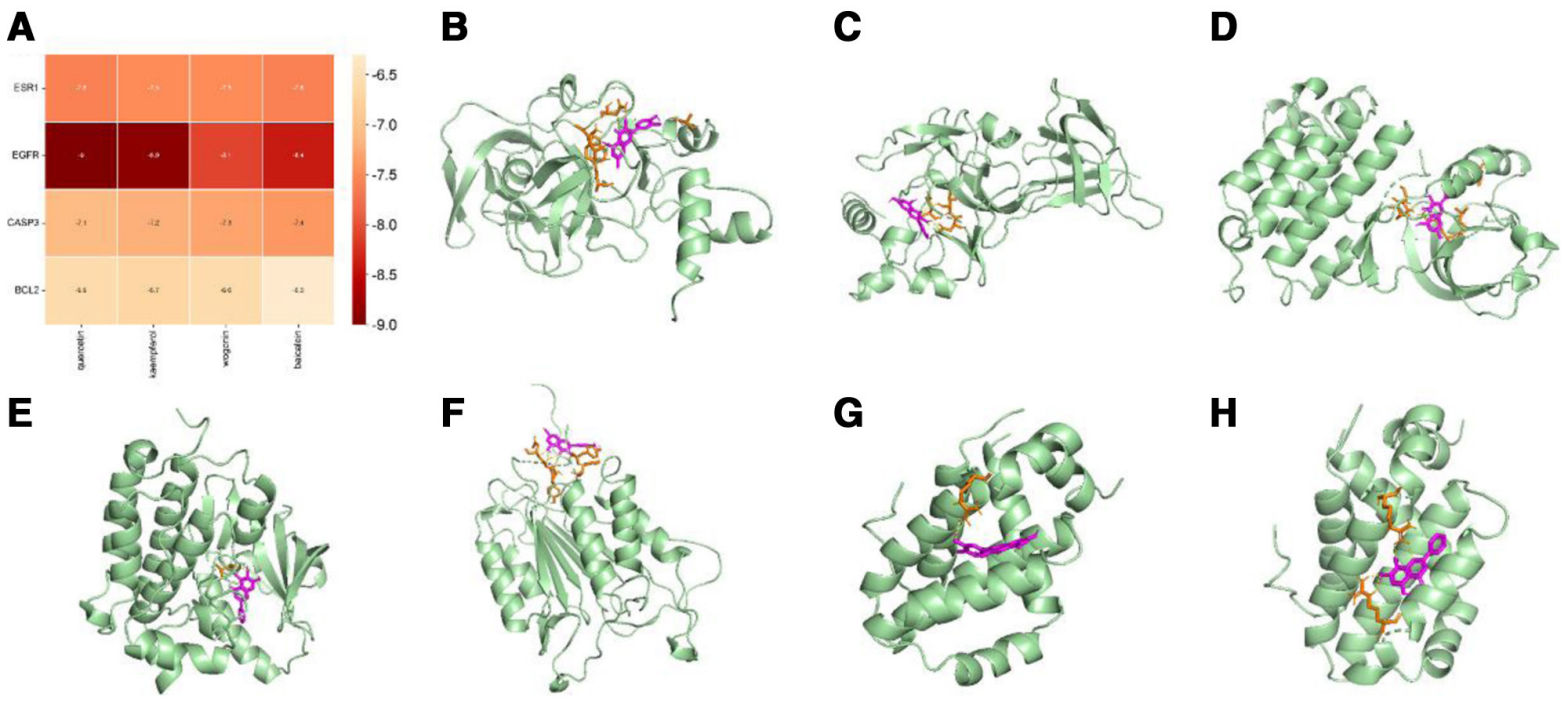
Molecular docking visualization diagram. (A) Molecular docking heatmap; (B) quercetin-ESR1; (C) kaempferol-ESR1; (D) quercetin-EGFR; (E) baicalein-EGFR; (F) quercetin-CASP3; (G) quercetin-BCL2; and (H) wogonin-BCL2. BCL2 = B-cell lymphoma-2, CASP3 = caspase-3, EGFR = epidermal growth factor receptor, ESR1 = estrogen receptor 1.

Based on the degree value, the top 4 core ingredients and key targets were selected as ligands and receptors. When ligands undergo molecular docking with receptors, a lower binding energy indicates superior binding activity. In this study, the docking binding energies of quercetin, kaempferol, wogonin, and baicalein with ESR1, EGFR, CASP3, and BCL2 were all below −5.0 kcal/mol (Fig. 7A). The results showed that quercetin was well docked to ESR1 (Fig. 7B), kaempferol to ESR1 (Fig. 7C), quercetin to EGFR (Fig. 7D), baicalein to EGFR (Fig. 7E), quercetin to CASP3 (Fig. 7F), quercetin to BCL2 (Fig. 7G), and wogonin to BCL2 (Fig. 7H). This indicates favorable docking interactions between the core ingredients of CGGD and key target proteins.

## 4. Discussion and analysis

STC combined with depression can be categorized as “constipation” and “depression” in Chinese medicine, and its basic pathology is the failure of conduction in the large intestine and the blockage of qi in the internal organs, which is mainly located in the large intestine, and is closely related to the internal organs of the liver, kidneys, lungs, spleen, stomach, etc. CGGD is composed of Chaihu, Guizhi, Ganjiang, *S baicalensis*, Gualougen, Calcined Oyster, and Zhigancao. The combination of all the herbs can achieve the efficacy of relaxing the qi, lubricating the intestines, and relieving the bowel movement.

In this study, we obtained the active ingredients and targets of CGGD and the targets of STC combined with depression through the TCMSP platform, UniProt database, GEO database, GeneCards database, and OMIM database, and intersected them to obtain 64 common targets. When screening key targets and core ingredients, the degree value directly reflects the connectivity breadth of the target or ingredient within the network. A higher degree value indicates more connected nodes, suggesting a broader potential scope for regulating disease-related pathways and a greater likelihood of being a core regulatory molecule. However, betweenness centrality and closeness centrality primarily characterize the bridging function and efficiency of signal transmission within networks, being more suited to network structural analysis rather than the screening of core regulatory molecules. Following screening, ESR1, EGFR, CASP3, BCL2, myelocytomatosis oncogene, IL6, hypoxia-inducible factor 1-alpha, G1/S-specific cyclin-D1, erb-B2 receptor tyrosine kinase 2, and peroxisome proliferator-activated receptor gamma were identified as key targets through which CGGD exerts its therapeutic effects. It has been revealed that the ESR1 gene participates in the human intestinal immune response.^[29]^ Xiong discovered that estrogen can inhibit colonic smooth muscle contraction in mice, thereby inducing constipation.^[30]^ The ESR1 gene is involved in the neurotransmitter transmission process in brain tissue after binding to estrogen receptor-α and affects the level of neurotrophic factor.^[31]^ Hu conducted a genome-wide association study, confirming that ESR1 polymorphisms are associated with gender-specific depression.^[32]^ EGFR regulates cell division, differentiation, and proliferation, and has antitumor activity.^[33]^ Research indicated that dural regulatory T cells may alleviate depression through EGFR signaling.^[34]^ CASP3, as a terminal shear enzyme in the process of apoptosis, has a promoting effect on apoptosis. Overexpression of CASP3 in cells not only severely damages the intestinal mucosal barrier, triggers defecation difficulties, and induces STC,^[35]^ but also leads to neuronal apoptosis, which participates in the process of depression.^[36]^ Dionisie V discovered that escitalopram exerts a neuroprotective effect on depression-related neurons by reducing the levels of overexpressed CASP3.^[37]^ BCL2 is an antiapoptotic protein capable of inhibiting cell death and enhancing cellular activity, thereby exerting a protective effect against STC.^[38]^ Furthermore, multiple studies have demonstrated that BCL2 levels in the hippocampus are markedly reduced in animal models of depression.^[39,40]^

Analysis of the active ingredients revealed that the top 10 ingredients with the highest degree values were quercetin, kaempferol, wogonin, baicalein, isorhamnetin, beta-sitosterol, licochalcone a, 7-methoxy-2-methyl isoflavone, naringenin, and acacetin, suggesting that these compounds may be the main active ingredients of CGGD for the treatment of STC combined with depression. Among the core ingredients, quercetin, kaempferol, wogonin, and baicalein belong to flavonoids, which have pharmacological effects such as anticancer, antioxidant, anti-inflammatory, and immunomodulatory effects.^[41,42]^ Studies have shown that quercetin can modulate gastrointestinal smooth muscle cells, restore intestinal motility, promote mucin secretion, and significantly increase fecal output in constipated rat models.^[43]^ Through network pharmacology and in vitro experiments, it was discovered that quercetin exerts antidepressant effects via monoaminergic neurotransmitters and cAMP signaling pathways, as well as neuroactive ligand-receptor interactions.^[44]^ Quercetin further exerts antidepressant effects by modulating the gut microbiota-gut-brain axis through regulating specific bacteria (such as romboutsia, turicibacter, faecalibaculum, and bifidobacterium), thereby restoring gut microbiota balance.^[45]^ Kaempferol, as one of the key ingredients for intervening in inflammation-related diseases, can suppress inflammatory levels and significantly alleviate inflammatory bowel disease and its induced constipation.^[46]^ Through in vitro and in vivo experiments, Chen demonstrated that kaempferol holds considerable potential in enhancing antioxidant capacity, reducing intestinal inflammation, and strengthening intestinal barrier function within gut physiology.^[47]^ Kaempferol also promoted the expression of BDNF and NGF proteins in hippocampal tissue of CUMS-induced depressive model rats, mitigating hippocampal damage in the rat brain and exerting neuroprotective and antidepressant effects.^[48,49]^ Furthermore, a systematic review and network meta-analysis indicated that kaempferol significantly ameliorates depressive-like behavior in rodents by exerting anti-inflammatory and antioxidant effects, alongside modulating levels of neurotrophic factors and neurotransmitters.^[50]^ Both wogonin and baicalein are flavonoids extracted from the roots of *S baicalensis*, family Labiatae. Wogonin can regulate cell growth, differentiation, and apoptosis, and has anti-inflammatory, antitumor, antioxidant, and neuroprotective effects.^[51]^ Wogonin is converted to baicalin in the human body, which can regulate the metabolism of intestinal flora,^[52]^ and also significantly reduces the expression levels of inflammatory factors IL-6 and IL-1β, improves the inflammatory response of the intestine, and maintains the intestinal endothelial homeostasis.^[53]^ Wogonin increases 5-HT and DA levels alongside BDNF optical density values in rat models of depression, promoting neurotrophic factor production and markedly alleviating depressive-like behavior in rats.^[54,55]^ Furthermore, researchers have demonstrated synergistic effects of wogonin in behavioral assessments, neurochemical analyses, and biochemical indicators across mouse models of depression, confirming its significant antidepressant potential.^[56]^ Baicalin exerts effects in regulating intestinal inflammation, relaxing colonic smooth muscle, and stabilizing the gut microbiota, thereby significantly improving intestinal function.^[57–59]^ Baicalin may also block the onset and progression of depression through diverse mechanisms, including neuronal protection, alleviation of neuroinflammation, regulation of the HPA axis, increased neurotransmitter expression, and reduction of cell apoptosis.^[60,61]^ The results of molecular docking showed that ESR1, EGFR, CASP3, and BCL2 had good affinity for quercetin, kaempferol, wogonin, and baicalein, suggesting that CGGD may exert its therapeutic effect in treating STC combined with depression by regulating the above targets.

From the results of GO analysis and KEGG pathway enrichment analysis, it can be seen that the treatment of STC combined with depression by CGGD involves a variety of molecular functions, a variety of BPs, and multiple pathways. Important BPs involved in CGGD include positive regulation of gene expression, negative regulation of the apoptotic process, signaling, etc, which affect protein binding, enzyme binding, transcription factor activity, and specific protein binding, etc, in the nucleus, cytoplasm, and cytoplasmic membrane. KEGG-enriched pathway suggests that CGGD may be involved in the regulation of STC combined with depression through PI3K-Akt signaling pathway, MAPK signaling pathway, ErbB signaling pathway, AGE-RAGE signaling pathway, apoptosis, and cancer pathway, and so on.

PI3K has serine/threonine kinase activity^[62]^ and can catalyze the conversion of phosphatidylinositol to phosphatidylinositol (3,4,5)-trisphosphate.^[63]^ Phosphatidylinositol (3,4,5)-trisphosphate can recruit Akt to the cell membrane for phosphorylation and activation. AKT (protein kinase B, PKB/Akt) is an important kinase that includes 3 isoforms, AKT1, AKT2, and AKT3, and plays a crucial role in the processes of cell proliferation, differentiation, and metabolism.^[64]^ Research has revealed that abnormal expression of the PI3K-Akt signaling pathway can cause damage to colonic tissue, potentially representing a key mechanism in the development of STC.^[65]^ Zhang demonstrated through animal studies that modulating the PI3K-Akt signaling pathway effectively alleviates 5-FU-induced constipation in mice.^[66]^ Findings from Wang et al further indicate sustained activation of the PI3K-Akt pathway within the complex laxative mechanisms of rhubarb and moringa leaf extracts.^[67]^ Additional research indicated that activation of the PI3K-Akt signaling pathway could downregulate reactive oxygen species expression levels in the cortex and hippocampus of rat models of depression, thereby reducing oxidative stress and enhancing neuroprotection.^[68]^ Furthermore, the PI3K-Akt signaling pathway may exert antidepressant effects by alleviating neuroinflammation, promoting neurogenesis, balancing neurotransmitters, and increasing synaptic plasticity.^[69]^

The MAPK signaling pathway contains a 3-tiered signaling process: the MAPK, the MAPK kinase (MEK or MKK), and the kinase of the MAPK kinase (MEKK or MKKK). The 3 kinases above are activated sequentially, which together regulate cellular These 3 kinases are activated sequentially, and together they regulate a variety of important physiological or pathological effects such as cell growth, differentiation, proliferation, and inflammatory response. It was found that Zengye Chengqi Decoction could improve intestinal peristalsis and restore intestinal transport function in rats by regulating the MAPK signaling pathway, down-regulating the expression of AQP3, and up-regulating the expression of AQP9.^[70]^ Another study showed that Zengye Decoction could promote interstitial cell of Cajal (ICCs) proliferation and inhibit apoptosis through the MAPK signaling pathway, and had a significant ameliorative effect on STC.^[71]^ Furthermore, Shan discovered that Glabridin exerts antidepressant effects through multiple mechanisms, including downregulating MAPK/NF-κB pathway activity, inhibiting inflammatory cytokine expression, and modulating neurotransmitters.^[72]^ The MAPK family is divided into 3 classes, namely extracellular signaling-associated kinases (ERKs), c-Jun N-terminal kinases (JNKs), and p38. ERKs are widely present in mammalian cells, and studies have shown that in depression patients and animal models, the expression of ERK signaling pathway molecules is significantly downregulated in both the prefrontal cortex and hippocampus, suggesting that the pathway molecules are involved in the disease progression of depression.^[73]^ The p38 MAPK pathway has been implicated in the pathogenesis of neuroinflammation and can attenuate monoamine neurotransmitter deficiencies and ameliorate depressive-like behaviors in mice by downregulating the expression of p38/NF-κB signaling pathway and inhibiting microglia-induced apoptosis.^[74]^ JNK is another subclass of the MAPK signaling pathway in mammalian cells, which can inhibit apoptosis in rat hippocampal neurons and play a protective role in the brain by decreasing the protein expression level of c-Jun, the main substrate of the rat hippocampal JNK signaling pathway, and reducing the expression of downstream apoptotic proteins.^[75]^ Research has also demonstrated that reducing the phosphorylation levels of ERK, JNK, and p38, thereby inhibiting the activation of the MAPK signaling pathway, can effectively alleviate symptoms of STC.^[76]^

Apoptosis refers to the genetically controlled, autonomous, and orderly death of cells in order to maintain the stability of the internal environment, and it plays an important role in the growth, development, and aging of the organism. ICCs, as a kind of intestinal motility pacemaker cell, are widely distributed in the smooth muscle of the whole digestive tract of the body, which is capable of generating slow waves to cause automatic rhythmic motility of smooth muscle in the gastrointestinal tract. Studies have shown that ICCs’ apoptosis in colonic tissues of the organism is closely related to the pathogenesis of STC, and inhibition of ICCs’ apoptosis is one of the important mechanisms for the treatment of STC.^[77–79]^ Wu experimental research revealed that the Bian-Se-Tong mixture significantly alleviates constipation symptoms in STC rats by activating the PI3K-Akt signaling pathway and improving the apoptosis of Cajal cells.^[80]^ Abnormal apoptosis, on the other hand, leads to dysregulation of neuroplasticity, which is closely related to the development of depression.^[81]^ Numerous studies have shown that the number of neuronal cell apoptosis in the hippocampal region is significantly elevated in depressed patients and CUMS-induced depression model rats, suggesting that apoptosis in the hippocampal region may be an important mechanism in the pathogenesis of depression, and that inhibition of neuronal apoptosis improves the symptoms of abnormal neurological function and alleviates the depressive state.^[82–84]^

## 5. Conclusions

In summary, this study employs network pharmacology and molecular docking techniques to preliminarily elucidate that CGGD regulates STC combined with depression through a complex, multi-ingredient, multi-target, multi-pathway, and nonlinear process. CGGD may exert therapeutic effects on STC combined with depression by acting on biological targets such as ESR1, EGFR, CASP3, and BCL2 through active ingredients including quercetin, kaempferol, wogonin, and baicalein. This occurs via intervention in multiple signaling pathways, including the PI3K-Akt pathway, MAPK pathway, and apoptosis pathway. Concurrently, this study presents certain challenges and limitations: firstly, network pharmacology analyses rely on existing database entries and lack direct experimental validation; secondly, molecular docking only verifies the in vitro binding potential between core ingredients and key targets, without addressing in vivo pharmacokinetic processes or the impact of synergistic or antagonistic interactions among compound constituents on therapeutic efficacy. Given these limitations, future research may explore the following avenues: firstly, establishing cellular and animal models to validate predictions through in vivo and in vitro experiments; secondly, investigating the in vivo metabolic mechanisms of core ingredients, combining pharmacokinetic studies to clarify absorption efficiency, metabolites, and interactions among compound constituents; thirdly, conducting small-scale clinical pilot studies to provide direct evidence for the precise clinical application of traditional Chinese medicine compounds.

## Acknowledgments

We are grateful to the Henan University of Chinese Medicine, the First Affiliated Hospital of Henan University of Chinese Medicine, and all participants in our study.

## Author contributions

**Conceptualization:** Zixu Zhao.

**Data curation:** Shuangxi Zhang.

**Formal analysis:** Lingfei Meng.

**Funding acquisition:** Shuangxi Zhang.

**Investigation:** Zixu Zhao, Chaolun Zhu, Lingfei Meng.

**Resources:** Chaolun Zhu, Lingfei Meng.

**Software:** Chaolun Zhu.

**Validation:** Zixu Zhao.

**Visualization:** Zixu Zhao.

**Writing – original draft:** Zixu Zhao.

**Writing – review & editing:** Shuangxi Zhang.






