# Supplementary material for: Study on the mechanism of action of Chaihu Guizhi Ganjiang Decoction for the treatment of slow transit constipation combined with depression based on network pharmacology and molecular docking
Source: Medicine (Baltimore). 2026 Jun 5;105(23):e49136. doi: 10.1097/MD.0000000000049136 (PMC13246040; doi:10.1097/MD.0000000000049136)
Supplement: Supplementary file 1 [file medi-105-e49136-s001.docx]

Supplementary Table 1. Information of 138 active ingredients of CGGD.

| MOL ID | MOL name | OB | DL | Source drugs |
| --- | --- | --- | --- | --- |
| MOL000098 | quercetin | 46.43 | 0.28 | Chaihu, Zhigancao |
| MOL000422 | kaempferol | 41.88 | 0.24 | Chaihu, Zhigancao |
| MOL000354 | isorhamnetin | 49.6 | 0.31 | Chaihu, Zhigancao |
| MOL000358 | beta-sitosterol | 36.91 | 0.75 | Chaihu, Ganjiang, Scutellaria Baicalensis |
| MOL000359 | sitosterol | 36.91 | 0.75 | Guizhi, Ganjiang, Scutellaria Baicalensis, Zhigancao |
| MOL000449 | Stigmasterol | 43.83 | 0.76 | Chaihu, Scutellaria Baicalensis |
| MOL000073 | ent-Epicatechin | 48.96 | 0.24 | Guizhi, Scutellaria Baicalensis |
| MOL001645 | Linoleyl acetate | 42.1 | 0.2 | Chaihu |
| MOL002776 | Baicalin | 40.12 | 0.75 | Chaihu |
| MOL004609 | Areapillin | 48.96 | 0.41 | Chaihu |
| MOL013187 | Cubebin | 57.13 | 0.64 | Chaihu |
| MOL004624 | LongikaurinA | 47.72 | 0.53 | Chaihu |
| MOL004598 | 3,5,6,7-tetramethoxy-2-(3,4,5-trimethoxyphenyl)chromone | 31.97 | 0.59 | Chaihu |
| MOL004718 | α-spinasterol | 42.98 | 0.76 | Chaihu |
| MOL000490 | petunidin | 30.05 | 0.31 | Chaihu |
| MOL001736 | (-)-taxifolin | 60.51 | 0.27 | Guizhi |
| MOL000492 | (+)-catechin | 54.83 | 0.24 | Guizhi |
| MOL004576 | taxifolin | 57.84 | 0.27 | Guizhi |
| MOL002464 | 1-Monolinolein | 37.18 | 0.3 | Ganjiang |
| MOL002501 | [(1S)-3-[(E)-but-2-enyl]-2-methyl-4-oxo-1-cyclopent-2-enyl] (1R,3R)-3-[(E)-3-methoxy-2-methyl-3-oxoprop-1-enyl]-2,2-dimethylcyclopropane-1-carboxylate | 62.52 | 0.31 | Ganjiang |
| MOL002514 | Sexangularetin | 62.86 | 0.3 | Ganjiang |
| MOL001689 | acacetin | 34.97 | 0.24 | Scutellaria Baicalensis |
| MOL000173 | wogonin | 30.68 | 0.23 | Scutellaria Baicalensis |
| MOL002714 | baicalein | 33.52 | 0.21 | Scutellaria Baicalensis |
| MOL002897 | epiberberine | 43.09 | 0.78 | Scutellaria Baicalensis |
| MOL008206 | Moslosooflavone | 44.09 | 0.25 | Scutellaria Baicalensis |
| MOL000228 | (2R)-7-hydroxy-5-methoxy-2-phenylchroman-4-one | 55.23 | 0.2 | Scutellaria Baicalensis |
| MOL002909 | 5,7,2,5-tetrahydroxy-8,6-dimethoxyflavone | 33.82 | 0.45 | Scutellaria Baicalensis |
| MOL002910 | Carthamidin | 41.15 | 0.24 | Scutellaria Baicalensis |
| MOL002913 | Dihydrobaicalin_qt | 40.04 | 0.21 | Scutellaria Baicalensis |
| MOL002914 | Eriodyctiol (flavanone) | 41.35 | 0.24 | Scutellaria Baicalensis |
| MOL002915 | Salvigenin | 49.07 | 0.33 | Scutellaria Baicalensis |
| MOL002917 | 5,2',6'-Trihydroxy-7,8-dimethoxyflavone | 45.05 | 0.33 | Scutellaria Baicalensis |
| MOL002925 | 5,7,2',6'-Tetrahydroxyflavone | 37.01 | 0.24 | Scutellaria Baicalensis |
| MOL002927 | Skullcapflavone II | 69.51 | 0.44 | Scutellaria Baicalensis |
| MOL002928 | oroxylin a | 41.37 | 0.23 | Scutellaria Baicalensis |
| MOL002932 | Panicolin | 76.26 | 0.29 | Scutellaria Baicalensis |
| MOL002933 | 5,7,4'-Trihydroxy-8-methoxyflavone | 36.56 | 0.27 | Scutellaria Baicalensis |
| MOL002934 | NEOBAICALEIN | 104.34 | 0.44 | Scutellaria Baicalensis |
| MOL002937 | DIHYDROOROXYLIN | 66.06 | 0.23 | Scutellaria Baicalensis |
| MOL000525 | Norwogonin | 39.4 | 0.21 | Scutellaria Baicalensis |
| MOL000552 | 5,2'-Dihydroxy-6,7,8-trimethoxyflavone | 31.71 | 0.35 | Scutellaria Baicalensis |
| MOL001458 | coptisine | 30.67 | 0.86 | Scutellaria Baicalensis |
| MOL002879 | Diop | 43.59 | 0.39 | Scutellaria Baicalensis |
| MOL010415 | 11,13-Eicosadienoic acid, methyl ester | 39.28 | 0.23 | Scutellaria Baicalensis |
| MOL012245 | 5,7,4'-trihydroxy-6-methoxyflavanone | 36.63 | 0.27 | Scutellaria Baicalensis |
| MOL012246 | 5,7,4'-trihydroxy-8-methoxyflavanone | 74.24 | 0.26 | Scutellaria Baicalensis |
| MOL012266 | rivularin | 37.94 | 0.37 | Scutellaria Baicalensis |
| MOL004355 | Spinasterol | 42.98 | 0.76 | Gualougen |
| MOL006756 | Schottenol | 37.42 | 0.75 | Gualougen |
| MOL001484 | Inermine | 75.18 | 0.54 | Zhigancao |
| MOL001792 | DFV | 32.76 | 0.18 | Zhigancao |
| MOL000211 | Mairin | 55.38 | 0.78 | Zhigancao |
| MOL002311 | Glycyrol | 90.78 | 0.67 | Zhigancao |
| MOL000239 | Jaranol | 50.83 | 0.29 | Zhigancao |
| MOL002565 | Medicarpin | 49.22 | 0.34 | Zhigancao |
| MOL003656 | Lupiwighteone | 51.64 | 0.37 | Zhigancao |
| MOL003896 | 7-Methoxy-2-methyl isoflavone | 42.56 | 0.2 | Zhigancao |
| MOL000392 | formononetin | 69.67 | 0.21 | Zhigancao |
| MOL000417 | Calycosin | 47.75 | 0.24 | Zhigancao |
| MOL004328 | naringenin | 59.29 | 0.21 | Zhigancao |
| MOL004805 | (2S)-2-[4-hydroxy-3-(3-methylbut-2-enyl)phenyl]-8,8-dimethyl-2,3-dihydropyrano[2,3-f]chromen-4-one | 31.79 | 0.72 | Zhigancao |
| MOL004806 | euchrenone | 30.29 | 0.57 | Zhigancao |
| MOL004808 | glyasperin B | 65.22 | 0.44 | Zhigancao |
| MOL004810 | glyasperin F | 75.84 | 0.54 | Zhigancao |
| MOL004811 | Glyasperin C | 45.56 | 0.4 | Zhigancao |
| MOL004814 | Isotrifoliol | 31.94 | 0.42 | Zhigancao |
| MOL004815 | (E)-1-(2,4-dihydroxyphenyl)-3-(2,2-dimethylchromen-6-yl)prop-2-en-1-one | 39.62 | 0.35 | Zhigancao |
| MOL004820 | kanzonols W | 50.48 | 0.52 | Zhigancao |
| MOL004824 | (2S)-6-(2,4-dihydroxyphenyl)-2-(2-hydroxypropan-2-yl)-4-methoxy-2,3-dihydrofuro[3,2-g]chromen-7-one | 60.25 | 0.63 | Zhigancao |
| MOL004827 | Semilicoisoflavone B | 48.78 | 0.55 | Zhigancao |
| MOL004828 | Glepidotin A | 44.72 | 0.35 | Zhigancao |
| MOL004829 | Glepidotin B | 64.46 | 0.34 | Zhigancao |
| MOL004833 | Phaseolinisoflavan | 32.01 | 0.45 | Zhigancao |
| MOL004835 | Glypallichalcone | 61.6 | 0.19 | Zhigancao |
| MOL004838 | 8-(6-hydroxy-2-benzofuranyl)-2,2-dimethyl-5-chromenol | 58.44 | 0.38 | Zhigancao |
| MOL004841 | Licochalcone B | 76.76 | 0.19 | Zhigancao |
| MOL004848 | licochalcone G | 49.25 | 0.32 | Zhigancao |
| MOL004849 | 3-(2,4-dihydroxyphenyl)-8-(1,1-dimethylprop-2-enyl)-7-hydroxy-5-methoxy-coumarin | 59.62 | 0.43 | Zhigancao |
| MOL004855 | Licoricone | 63.58 | 0.47 | Zhigancao |
| MOL004856 | Gancaonin A | 51.08 | 0.4 | Zhigancao |
| MOL004857 | Gancaonin B | 48.79 | 0.45 | Zhigancao |
| MOL004863 | 3-(3,4-dihydroxyphenyl)-5,7-dihydroxy-8-(3-methylbut-2-enyl)chromone | 66.37 | 0.41 | Zhigancao |
| MOL004864 | 5,7-dihydroxy-3-(4-methoxyphenyl)-8-(3-methylbut-2-enyl)chromone | 30.49 | 0.41 | Zhigancao |
| MOL004866 | 2-(3,4-dihydroxyphenyl)-5,7-dihydroxy-6-(3-methylbut-2-enyl)chromone | 44.15 | 0.41 | Zhigancao |
| MOL004879 | Glycyrin | 52.61 | 0.47 | Zhigancao |
| MOL004882 | Licocoumarone | 33.21 | 0.36 | Zhigancao |
| MOL004883 | Licoisoflavone | 41.61 | 0.42 | Zhigancao |
| MOL004884 | Licoisoflavone B | 38.93 | 0.55 | Zhigancao |
| MOL004885 | licoisoflavanone | 52.47 | 0.54 | Zhigancao |
| MOL004891 | shinpterocarpin | 80.3 | 0.73 | Zhigancao |
| MOL004898 | (E)-3-[3,4-dihydroxy-5-(3-methylbut-2-enyl)phenyl]-1-(2,4-dihydroxyphenyl)prop-2-en-1-one | 46.27 | 0.31 | Zhigancao |
| MOL004903 | liquiritin | 65.69 | 0.74 | Zhigancao |
| MOL004904 | licopyranocoumarin | 80.36 | 0.65 | Zhigancao |
| MOL004907 | Glyzaglabrin | 61.07 | 0.35 | Zhigancao |
| MOL004908 | Glabridin | 53.25 | 0.47 | Zhigancao |
| MOL004910 | Glabranin | 52.9 | 0.31 | Zhigancao |
| MOL004911 | Glabrene | 46.27 | 0.44 | Zhigancao |
| MOL004912 | Glabrone | 52.51 | 0.5 | Zhigancao |
| MOL004913 | 1,3-dihydroxy-9-methoxy-6-benzofurano[3,2-c]chromenone | 48.14 | 0.43 | Zhigancao |
| MOL004914 | 1,3-dihydroxy-8,9-dimethoxy-6-benzofurano[3,2-c]chromenone | 62.9 | 0.53 | Zhigancao |
| MOL004915 | Eurycarpin A | 43.28 | 0.37 | Zhigancao |
| MOL004924 | (-)-Medicocarpin | 40.99 | 0.95 | Zhigancao |
| MOL004935 | Sigmoidin-B | 34.88 | 0.41 | Zhigancao |
| MOL004945 | (2S)-7-hydroxy-2-(4-hydroxyphenyl)-8-(3-methylbut-2-enyl)chroman-4-one | 36.57 | 0.32 | Zhigancao |
| MOL004948 | Isoglycyrol | 44.7 | 0.84 | Zhigancao |
| MOL004949 | Isolicoflavonol | 45.17 | 0.42 | Zhigancao |
| MOL004957 | HMO | 38.37 | 0.21 | Zhigancao |
| MOL004959 | 1-Methoxyphaseollidin | 69.98 | 0.64 | Zhigancao |
| MOL004961 | Quercetin der. | 46.45 | 0.33 | Zhigancao |
| MOL004966 | 3'-Hydroxy-4'-O-Methylglabridin | 43.71 | 0.57 | Zhigancao |
| MOL000497 | licochalcone a | 40.79 | 0.29 | Zhigancao |
| MOL004974 | 3'-Methoxyglabridin | 46.16 | 0.57 | Zhigancao |
| MOL004978 | 2-[(3R)-8,8-dimethyl-3,4-dihydro-2H-pyrano[6,5-f]chromen-3-yl]-5-methoxyphenol | 36.21 | 0.52 | Zhigancao |
| MOL004980 | Inflacoumarin A | 39.71 | 0.33 | Zhigancao |
| MOL004985 | icos-5-enoic acid | 30.7 | 0.2 | Zhigancao |
| MOL004988 | Kanzonol F | 32.47 | 0.89 | Zhigancao |
| MOL004989 | 6-prenylated eriodictyol | 39.22 | 0.41 | Zhigancao |
| MOL004990 | 7,2',4'-trihydroxy－5-methoxy-3－arylcoumarin | 83.71 | 0.27 | Zhigancao |
| MOL004991 | 7-Acetoxy-2-methylisoflavone | 38.92 | 0.26 | Zhigancao |
| MOL004993 | 8-prenylated eriodictyol | 53.79 | 0.4 | Zhigancao |
| MOL004996 | gadelaidic acid | 30.7 | 0.2 | Zhigancao |
| MOL000500 | Vestitol | 74.66 | 0.21 | Zhigancao |
| MOL005000 | Gancaonin G | 60.44 | 0.39 | Zhigancao |
| MOL005001 | Gancaonin H | 50.1 | 0.78 | Zhigancao |
| MOL005003 | Licoagrocarpin | 58.81 | 0.58 | Zhigancao |
| MOL005007 | Glyasperins M | 72.67 | 0.59 | Zhigancao |
| MOL005008 | Glycyrrhiza flavonol A | 41.28 | 0.6 | Zhigancao |
| MOL005012 | Licoagroisoflavone | 57.28 | 0.49 | Zhigancao |
| MOL005016 | Odoratin | 49.95 | 0.3 | Zhigancao |
| MOL005017 | Phaseol | 78.77 | 0.58 | Zhigancao |
| MOL005018 | Xambioona | 54.85 | 0.87 | Zhigancao |
| MOL005020 | dehydroglyasperins C | 53.82 | 0.37 | Zhigancao |
|  | Aluminum |  |  | Calcined Oyster |
|  | Calcium Sulphate |  |  | Calcined Oyster |
|  | Calcium Phosphate |  |  | Calcined Oyster |
|  | Silicon |  |  | Calcined Oyster |
|  | Calcium Carbonate |  |  | Calcined Oyster |
