# Supplementary material for: Study on the mechanism of action of Chaihu Guizhi Ganjiang Decoction for the treatment of slow transit constipation combined with depression based on network pharmacology and molecular docking
Source: Medicine (Baltimore). 2026 Jun 5;105(23):e49136. doi: 10.1097/MD.0000000000049136 (PMC13246040; doi:10.1097/MD.0000000000049136)
Supplement: Supplementary file 2 [file medi-105-e49136-s002.docx]

Supplementary Table 2. Information of 141 drug targets of CGGD.

| PTGS1 | NCOA2 | PGR | NR3C2 | NCOA1 |
| --- | --- | --- | --- | --- |
| AKR1B1 | PLAU | CTRB1 | CHRM3 | CHRM1 |
| ADRA1A | CHRM2 | GABRA1 | ESR1 | AR |
| PPARG | ESR2 | GSK3B | PRSS1 | CHEK1 |
| F7 | ACHE | GRIA2 | RELA | OLR1 |
| IKBKB | BCL2 | AHSA1 | CASP3 | MAPK8 |
| CYP3A4 | CYP1A1 | ICAM1 | SELE | VCAM1 |
| CYP1B1 | ALOX5 | GSTP1 | AHR | PSMD3 |
| SLC2A4 | NR1I3 | DIO1 | GSTM1 | GSTM2 |
| AKR1C3 | EGFR | VEGFA | CCND1 | FOS |
| EIF6 | CASP9 | RB1 | IL6 | TP63 |
| ELK1 | NFKBIA | POR | CASP8 | RAF1 |
| PRKCA | HIF1A | RUNX1T1 | ERBB2 | ACACA |
| CAV1 | MYC | PTGER3 | BIRC5 | DUOX2 |
| NOS3 | HSPB1 | MGAM | CCNB1 | NFE2L2 |
| NQO1 | PARP1 | COL3A1 | DCAF5 | CHEK2 |
| HSF1 | CRP | RUNX2 | RASSF1 | CTSD |
| IGFBP3 | IGF2 | IRF1 | ERBB3 | PON1 |
| NPEPPS | HK2 | RASA1 | CHRM4 | CHRNA2 |
| MTTP | APOB | FASN | TEP1 | MCL1 |
| FOSL1 | CYCS | NOX5 | APOD | CACNA2D1 |
| CYP2C9 | PDE10A | HTR3A | CHRM5 | MAPK10 |
| LDLR | SREBF1 | ABCC1 | AKR1C1 | ABAT |
| RXRB | TF | ATP1A1 | CACNB1 | CACNA1S |
| CACNA1C | CACNB2 | CACNG1 | HTR2A | FOXL2 |
| DMTN | KCNMA1 | P2RX2 | P2RX3 | CHRNA3 |
| CHRNB2 | CHRNB4 | SLC12A4 | SLC12A2 | SLC12A1 |
| SLC12A7 | SLC12A5 | SLC12A6 | SLC18A2 | HTR1A |
| FDPS |  |  |  |  |
