# Supplementary material for: Study on the mechanism of action of Chaihu Guizhi Ganjiang Decoction for the treatment of slow transit constipation combined with depression based on network pharmacology and molecular docking
Source: Medicine (Baltimore). 2026 Jun 5;105(23):e49136. doi: 10.1097/MD.0000000000049136 (PMC13246040; doi:10.1097/MD.0000000000049136)
Supplement: Supplementary file 3 [file medi-105-e49136-s003.docx]

Supplementary Table 3. Information of 64 co-targets of CGGD and STC combined with depression.

| PTGS1 | PGR | AKR1B1 | PLAU | CHRM3 |
| --- | --- | --- | --- | --- |
| GABRA1 | ESR1 | AR | PPARG | GSK3B |
| CHEK1 | ACHE | RELA | BCL2 | CASP3 |
| MAPK8 | CYP3A4 | CYP1A1 | ICAM1 | CYP1B1 |
| GSTP1 | GSTM1 | EGFR | VEGFA | CCND1 |
| FOS | CASP9 | RB1 | IL6 | TP63 |
| NFKBIA | CASP8 | RAF1 | PRKCA | HIF1A |
| ERBB2 | CAV1 | MYC | BIRC5 | DUOX2 |
| HSPB1 | CCNB1 | NFE2L2 | NQO1 | PARP1 |
| CHEK2 | CRP | RUNX2 | RASSF1 | CTSD |
| IGFBP3 | IGF2 | ERBB3 | APOB | CYCS |
| CYP2C9 | HTR3A | MAPK10 | SREBF1 | TF |
| HTR2A | SLC12A2 | SLC12A1 | SLC12A6 |  |
